# The Physiological Function and Potential Role of the Ubiquitous Na^+^/H^+^ Exchanger Isoform 8 (NHE8): An Overview Data

**DOI:** 10.3390/ijms231810857

**Published:** 2022-09-17

**Authors:** Claudio Bernardazzi, Irshad Ali Sheikh, Hua Xu, Fayez K. Ghishan

**Affiliations:** Department of Pediatrics, Steele Children’s Research Center, The University of Arizona, Tucson, AZ 85721, USA

**Keywords:** NHE8, eyes, kidney, testis, gastrointestinal tract, lungs

## Abstract

The Na^+^/H^+^ exchanger transporters (NHE) play an important role in various biologic processes including Na^+^ absorption, intracellular pH homeostasis, cell volume regulation, proliferation, and apoptosis. The wide expression pattern and cellular localization of NHEs make these proteins pivotal players in virtually all human tissues and organs. In addition, recent studies suggest that NHEs may be one of the primeval transport protein forms in the history of life. Among the different isoforms, the most well-characterized NHEs are the Na^+^/H^+^ exchanger isoform 1 (NHE1) and Na^+^/H^+^ exchanger isoform 3 (NHE3). However, Na^+^/H^+^ exchanger isoform 8 (NHE8) has been receiving attention based on its recent discoveries in the gastrointestinal tract. In this review, we will discuss what is known about the physiological function and potential role of NHE8 in the main organ systems, including useful overviews that could inspire new studies on this multifaceted protein.

## 1. Introduction

The presence of the electroneutral Na^+^/H^+^ exchanger (NHE) was first proposed by Mitchell in 1966 to describe the exchange of cations and protons across the mitochondrial membrane [1]. The NHE family has different isoforms, and it is identified in all domains of life, suggesting a very early role in evolution. In fact, recent studies suggest that NHEs may be one of the first primeval transport protein forms in the history of life [2]. Given the primitive origin of these NHEs transporters, there is a huge diversity of NHE across various realms thus emphasizing the wide expression pattern, the cellular localization, and the pivotal role they play in virtually all human tissues and organs [2].

The NHE family exploits the electrochemical Na^+^ concentration gradients that are developed across the cell membrane due to secondary active transport of Na^+^/K^+^/ATPase, participating in the regulation of various fundamental biologic processes including Na^+^ absorption, intracellular pH homeostasis, cell volume regulation, proliferation, and apoptosis. Alteration of NHE function is also implicated in various pathophysiological conditions. Autosomal recessive loss-of-function mutation in the Na^+^/H^+^ exchanger isoform 3 (NHE3)-encoding solute carrier family 9 member A3 (SLC9A3) gene has been linked to the pathogenesis of congenital sodium diarrhea characterized by high fecal loss of Na^+^ [3]. Furthermore, evidence has been shown that mutations in the isoform 6 or 9 of the NHE genes cause X-linked mental retardation and familial autism, respectively [4,5].

**Figure 1:**
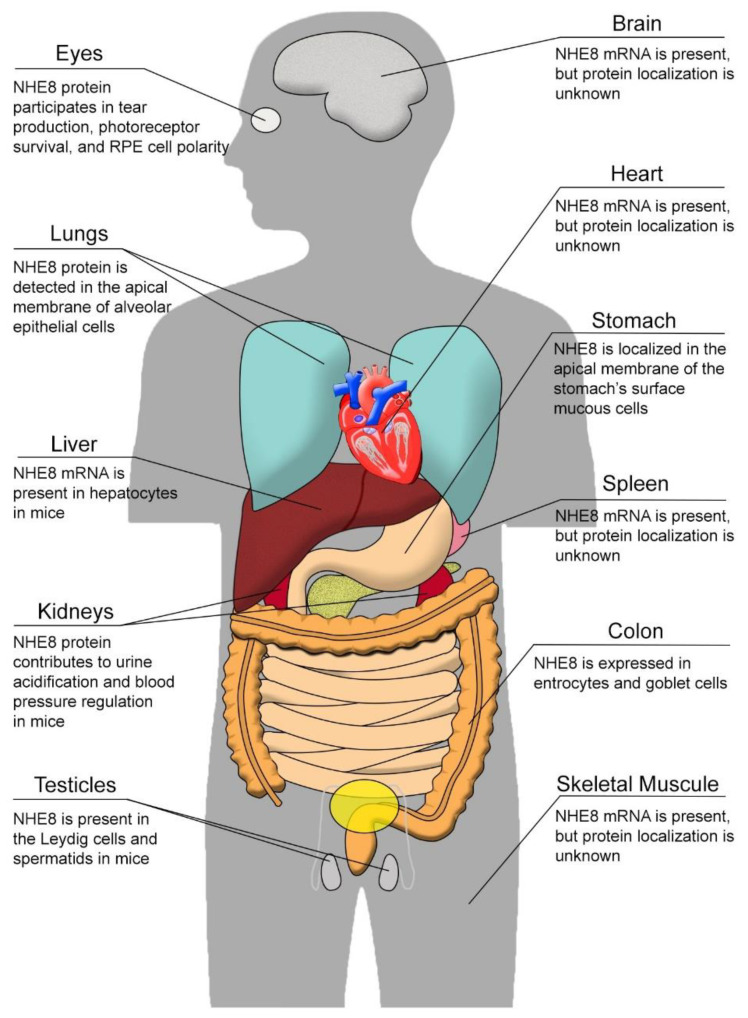
Scheme summarizing the expression of NHE8 in different organs.

Na^+^/H^+^ exchanger isoform 8 (NHE8), encoded by the SLC9A8 gene, is ubiquitously expressed in many tissues. NHE8 protein has been detected in the plasma membrane, Golgi apparatus, and endosomes in different cell types [6,7]. Akin to other NHEs in the NHE family, NHE8 is involved in electroneutral NaCl transport [8]. However, the function of NHE8 extends beyond simple Na^+^/H^+^ exchanging. NHE8 also participates in cell volume regulation, cellular adhesion, migration, and proliferation [9]. In addition, NHE8 plays a compensatory role in the absence of other NHE isoforms. In this review paper, we will discuss what is known about the physiological function and potential role of NHE8 since it was discovered. We will also focus on its tissue expression, cellular localization, and unique function in various tissues as summarized in Figure 1, briefly introducing the other NHE isoforms and their roles in each relevant tissue.

### 1.1. NHEs Classification

As per the most current classification, NHEs are part of the cation proton antiporter (CPA) superfamily that can be further subdivided into CPA1, CPA2, and CPA3 [10,11]. The largest among the three, the CPA1 superfamily, is comprised of three subfamilies: SLC9A1–9 (NHE1–9), SLC9B1–2 (NHA1–2), and SLC9C1–2 (NHE10-11, the so-called sperm NHE, and an orphan-related protein). It also includes transporter proteins from bacteria, archaea, yeast, plants, and mammals. The CPA1 superfamily differs in terms of substrate preferences, kinetics, and tissue localizations [12]. More information about the CPA1 superfamily can be found in Table 1.

### 1.2. The Molecular Structure of NHEs

**Figure 2:**
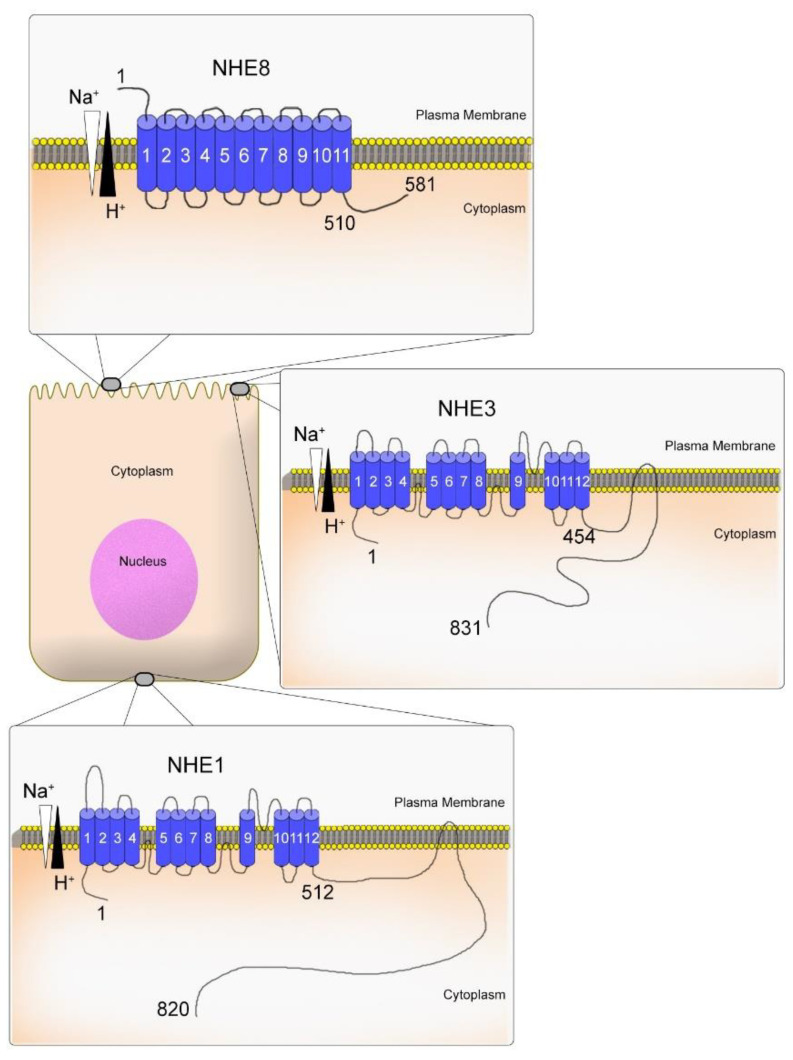
Scheme representing the molecular structure of NHE isoform 1 (NHE1), NHE isoform 3 (NHE3), and NHE isoform 8 (NHE8) in the plasma membrane. Data are based on the information from the NCBI website accessed on 1 September 2022 (https://www.ncbi.nlm.nih.gov/nuccore). This illustration was inspired by Orlowski and Grinstein, 2004.

The primary structure of NHE (NHE isoform 1–NHE1) was first discovered by Sardet et al. in 1989 using a genetic complementation approach [45]. Subsequently, other studies discovered more distant related NHE genes that were identified and characterized partially [17,46,47,48]. The molecular structure of NHE8 is still not well known. However, based on the primary structure of NHEs, all isoforms contain a highly conserved 10–12 transmembrane domain that mediates Na^+^/H^+^ exchange. The N-terminus region contains approx. 450 amino acids that may be cleaved during protein maturation. The hydrophilic C-terminus region that faces the cytoplasm contains a more divergent domain among the isoforms with a varying length between 70–440 amino acids [46,49]. For example, the two NHE isoforms well studied, NHE1 and NHE3, present a long C-terminus region with 308 amino acids and 377 amino acids, respectively [46]. These long regions have protein interactive sites to bind with multiple phosphorylation sites [46]. However, the NHE8 C-terminus region is short, containing around 70 amino acids with only three phosphorylation sites (Figure 2). According to the UniProt database, NHE8 may interact with 29 proteins [19]. However, none of these protein-protein interactions have been confirmed experimentally. A more detailed molecular structure of the NHE family can be found in the review paper published by John Orlowski and Sergio Grinstein [46].

## 2. NHE8 in the Gastrointestinal Tract

The intestinal epithelia play important roles in barrier function and nutrient absorption. The NHE family is a major player in electroneutral sodium absorption. NHE2, 3, and 8 are expressed in the apical membrane of the intestinal epithelial cells with distinctive patterns. The expression of NHE8 is shown throughout the entire gastrointestinal tract, with high expression detected in the jejunum and colon in both humans and mice [9].

The function of NHE8 was characterized by Xu et al., via transfecting the rat NHE8 cDNA into the Na^+^/H^+^ exchanger deficient PS120 cells. It was shown that NHE8 has a relatively higher sodium affinity than NHE2 and a similar proton affinity as NHE3 [9]. Regarding the NHE inhibitor sensitivity, NHE8 displays concentration-dependent sensitivity to HOE694 and S3226 [9]. As a physiological modulator, EGF is known to accelerate intestinal maturation and enhance sodium absorption. While EGF stimulates the activity of NHE2 and NHE3 [50,51], EGF affects NHE8 expression depending on the segment of the intestine. In a study performed in 2010, Xu et al. showed that EGF reduces the endogenous NHE8 gene expression in the ileum of rats (but not in the jejunum) [34]. Mechanistic studies showed that NHE8 inhibition by EGF was mediated by reducing the basal promoter activation of NHE8 [34,52]. In addition, methylprednisolone, a glucocorticoid involved in many physiological processes such as acceleration of intestinal epithelial maturation and enhanced sodium absorption, was shown to inhibit the gene expression and protein abundance of NHE8 in the small intestine of young rats [52].

### 2.1. The Gender-Specific Compensation in the Intestine

Among the NHE isoforms identified, three (NHE2, 3, and 8) have a significant role in the intestine. NHE2 was initially studied as an NHE isoform responsible for intestinal sodium absorption. However, no significant alteration could be observed in the intestine in the absence of NHE2 [18]. On the other hand, in the absence of NHE3, impaired sodium absorption results in mild diarrhea [53]. Interestingly, NHE3 and NHE2 do not have compensatory roles in the loss of each other [54]. However, NHE8 can compensate for the loss of either NHE2 or NHE3 in a gender-dependent manner. Under normal physiological conditions, the expression of NHE8 in the small intestine is lower in adult male mice compared with adult female mice. In NHE2X3 DKO mice, female NHE2X3 DKO mice have higher survival rates than male NHE2X3 DKO mice. This may be due to the increased NHE8 expression in female NHE2X3 DKO mice. When Caco-2 cells were treated with estradiol or testosterone, the expression of NHE8 was not altered by estradiol treatment, but testosterone treatment led to a significant reduction of NHE8 at both the gene expression and protein level, suggesting a gender-specific regulation is involved in NHE8 expression regulation in the intestine [55].

### 2.2. NHE8: The Bridge NHE Isoform for the Secretory Lineage?

The intestine is composed of two lineages of cells that are constantly renewed by the leucine-rich repeat-containing G-protein coupled receptor (Lgr5) positive intestinal stem cells: the absorptive and the secretory lineages. Most cell types in the intestine are enterocytes (~85%), which are derived from absorptive lineage. On the other hand, the secretory lineage is represented by goblet cells (~5%), Paneth cells (~5%), enteroendocrine cells (~1%), and tuft cells (<1%) [56,57]. The differentiation and maturation occur from the crypt to the villus, and it is regulated by various signaling pathways. Any breakdown that could cause an imbalance in these cell populations may be implicated in the pathogenesis of inflammatory bowel diseases (IBD) [58].

One of the most studied signaling pathways involved in the developmental process that regulates the fate of intestinal epithelial cells is the Notch pathway [59]. When Notch is stimulated by its ligand, Delta-like Ligand (DLL) or Jagged, it triggers a proteolytic cleavage mediated by _γ_-secretase that generates a Notch Intracellular domain (NICD). NICD is translocated to the nucleus and promotes gene transcription. One of the transcriptional factors activated by Notch is Hes1, which stimulates the absorptive lineage in the intestine. Hes1 also acts as an inhibitor of AtoH1 (MATH1), which is involved in the stimulation of secretory lineage. When cells migrate out of the Notch pathway, they start to increase the expression of AtoH1 and differentiate toward the secretory lineage [60]. It was shown that loss of NHE8 expression led to reduced mucin production via reduced goblet cells as well as reduced Paneth cell numbers [61,62]. This observation could be associated with the modulation of HES1 and ATOH1 expression levels. However, it must be investigated.

### 2.3. Lack of NHE8 as a Cause of Dysbiosis

The gut is colonized by trillions of bacteria distributed throughout the gastrointestinal tract that is shaped by diet, the immune system, and genetic factors of the host [63]. It is critical for the organism to be able to identify whether a stimulus is pathogenic or not to respond properly and maintain homeostasis.

Muc2 is a key component of the mucus layer. Without Muc2, the mucin layer that coats the intestinal epithelial cells and separates from luminal contents will be compromised, which would favor the proximity of the commensal and pathogenic microbes, stimulating an intestinal inflammatory response. It has been shown that mice lacking NHE8 have reduced Muc2 expression [61], and they were prone to bacterial adhesion and penetration [62].

Further studies showed that NHE8KO mice also developed dysbiosis [64], which is characterized by the increase of Bacteroidetes and the decrease of Firmicutes, especially the genus *Butyricimonas* and genus *Ruminiclostridium_5* and *_6* [65]. In a fecal material transfer study, the composition of microbiota in NHE8KO mice colonized with fecal materials from healthy wild-type control mice was altered with an increase of Bacteroidetes, Lactobacillus, and segmented filamentous bacteria (SFB). Treatment with fecal material from wild-type mice or probiotics is not enough to restore the bacterial population in NHE8KO mice and/or restore mucin production [65]. These observations suggest that NHE8 plays an important role in modulating bacterial diversity [65].

### 2.4. The Relationship between NHE8 and Diarrhea

The GI tract is lined with various types of ion channels and transporters that function to selectively absorb many nutrients and electrolytes across the intestinal epithelia while flushing toxic chemicals and antigens by maintaining intestinal lumen hydration [66,67]. Any alteration in this dynamic balance between fluid and electrolyte absorption and secretion often leads to diarrhea where either absorption is inhibited, secretion is enhanced or, in a few cases, both processes are affected [68].

Apart from NHE3, NHE8 plays an important role in water and electrolyte transport across the epithelia [69]. In fact, octreotide, an analog of somatostatin has been used as an anti-diarrheal drug for decades [70]. The study by Wang et al. demonstrated that somatostatin, a neuropeptide produced by intestinal D cells, specifically stimulates NHE8 but not NHE3 expression in mouse intestines, probably via the SSTR2-p38 MAPK pathway [71]. Moreover, studies in DSS-induced colitis and *Citrobacter rodentium*-infected colitis mouse models strongly suggest that upregulation of NHE8 by somatostatin ameliorates diarrheal symptoms in these colitis mouse models [72,73].

### 2.5. The Relationship between NHE8 and Colitis-Associated Colorectal Cancer

The first line of defense against harmful intestinal luminal contents is the continuously renewing epithelial cells and the inner and outer mucin layer produced by goblet cells [74]. The maintenance of a protective barrier against harmful intestinal luminal contents is pivotal for intestinal homeostasis, and the control of cell renewal depends on the equilibrium between cell proliferation and death. Any imbalance between the two mechanisms is known to result in colorectal cancer (CRC) [75,76].

The majority of human CRC are believed to originate from the activation of multiple signaling pathways, including Wnt signaling, mutation of the APC or β-catenin genes, Hedgehog signaling, p53, and others [77,78,79]. Chronic inflammation is also believed to be involved in the development of CRC [80]. The dysfunction of ion channels, including NHE isoforms, is involved in abnormal proliferation, invasion, and apoptosis, which could lead to CRC [81,82]. For example, NHE1 upregulation in cancer cells stimulates cell proliferation, migration, invasion, and apoptosis, and leads to an intracellular alkalinization and an extracellular acidic tumor environment [83]. NHE3 inhibition is associated with DNA damage and increased local inflammation [84]. In the absence of NHE8, mice have been shown to be susceptible to a colitis-inducible model, dysbiosis, and hyperproliferation [62]. In addition, NHE8 expression is downregulated in colitis animal models and in human UC, and the expression of NHE8 is absent in human colorectal tumors [62,64,85]. NHE8-deficient mice show hyperproliferation and high tumor incidence in colitis-associated CRC via increased expression of Lgr5, β-catenin, and c-Myc [86]. All the data suggested the important roles of NHE8 in intestinal homeostasis and how it contributes to the pathogenesis of colitis-associated CRC.

## 3. The Role of NHE8 in the Kidney

The kidney is an essential organ responsible for removing waste, controlling electrolytes, and acid-base balance. The structural and functional unit of the kidney is the nephron. Filtration of the blood occurs in the glomerulus, which is wrapped by the Bowman’s capsule in the proximal tubule. Under normal physiological conditions, two-thirds of filtered salt, water, and bicarbonate are reabsorbed in the proximal tubule; pointing to the involvement of ion transporters in the kidney [87].

NHE3 is the most studied NHE isoform involved in proton secretion across the apical membrane of the proximal tubule. However, NHE8 has been shown to be expressed in the same region as NHE3 in the kidney [88]. The function of NHEs in the kidney is further identified using mutant mouse models. NHE2KO mice present similar rates of Na^+^-dependent proton secretion compared to wild-type mice. On the other hand, NHE3KO mice present approximately 50% Na^+^-dependent proton secretion compared to wild-type mice. In NHE3KO/NHE2KO mice, the rate of Na^+^-dependent secretion was the same as that of NHE3KO mice. These observations suggest the existence of another NHE isoform in the proximal tubule participating in acid-base homeostasis regulation [89].

NHE8 was first discovered in the kidney by Sunita Goyal et al. in 2003, as a novel NHE isoform that mediates apical membrane ion transport in the proximal tubules in rats [90]. The same group also showed that neonatal rats have a higher expression level of NHE8 in the brush border membrane vesicles which is decreased 26 days after birth, a pattern opposite to the expression level of NHE3 [88]. Further study suggested that this developmental change of NHE8 expression is mediated by thyroid hormone [91]. In neonatal proximal tubules, metabolic acidosis results in the increased expression of both NHE8 and NHE3 isoforms [92,93]. The acidosis induced NHE8 activity increase was further confirmed in NRK cells [94,95].

In a study using mutant NHE3 and/or NHE8 mouse models, Michel Baum et al., showed that NHE3 protein abundance was increased in the absence of NHE8. Interestingly, the authors observed that serum bicarbonate levels were quite similar between NHE3KO/NHE8KO mice and NHE3KO mice, but the activity of NHE and blood pressure were lower in NHE3KO/NHE8KO mice compared with NHE3KO mice. These observations suggest that NHE8 may play a role in blood pressure regulation [96].

## 4. NHE8 Expression in the Eyes

NHE8 is highly expressed in the ocular tissue in humans and mice. It plays important roles in protecting the ocular surface. In the absence of NHE8, the tear volume is reduced by approximately 34%, along with increased focal inflammatory infiltration and foamy vacuoles within acinar cells in the lacrimal gland [7]. The conjunctival epithelial layer may also contribute to the reduction of tear volume since abnormal expression of small proline-rich protein 2H (Sprr2h) and transglutaminase 1 (Tgm1) were detected in the eyes of NHE8KO mice [97]. MUC5AC, primarily secreted mucin involved in the lubrication of the ocular surface, is produced by the goblet cells in the conjunctiva [97]. In the absence of NHE8, the expression of Muc5ac is reduced by 60%, suggesting a role of NHE8 in goblet cell function in the eye [97].

The eye is composed of the cornea, iris, pupil, lens, conjunctiva, retina, and other parts to gather light and produce an image that will be transmitted by the optic nerve to the brain. One of the mechanisms that protect the eye against harmful agents is the stimulation of tears by lacrimal glands and conjunctiva to lubricate the ocular surface. Any alteration that leads to the malfunction of tear production may lead to an inflammatory response, which may contribute to the development of dry eye disease. Dry eye is a multifactorial disease that results in tear film instability, discomfort, and visual disturbance [98].

In the back of the eye is the retina. The retinal pigment epithelium (RPE), a monolayer of polarized cells, maintains the homeostasis of the photoreceptor cells [99]. A study performed in rodents showed that an NHE8 mutant led to a resistance of photoreceptor cell death via regulating RPE cell polarity and function. The authors hypothesized that NHE8 protein could regulate pH homeostasis in the protein trafficking pathway [7]. In fact, in a posterior study using the AAV-CRISPR-Cas9 gene editing system to knock down NHE8 in mature retina, the same group showed that NHE8 proteins are localized in the inner segments of the photoreceptor. NHE8 plays a role in photoreceptor survival and in the maintenance of intracellular pH homeostasis in RPE cells [100]. A similar result was also observed by another group showing that mice lacking NHE8 have a noticeable ocular dysfunction, with retinal degeneration and immune cell infiltration associated with abnormal recycling endosomes in the RPE [6].

## 5. NHE8 in the Testis: Another Fertility Related NHE Isoform

According to the international glossary on infertility and fertility care, fertility is the capacity to establish a clinical pregnancy after one year of regular unprotected sexual intercourse. On the other hand, infertility is a disability to procreation. A third term, subfertility, can be used interchangeably with infertility, and it is related to reduced fertility in unsuccessful sexual intercourse with an aim of procreation [101]. Approximately one in seven couples are infertile, a condition that is well-recognized by the WHO [102]. The etiology of male infertility can vary from genetic mutations, diseases, and/or unhealthy lifestyles. However, testicular dysfunction is the most frequent cause that affects male fertility [103].

Among the five NHE isoforms detected in the testis (NHE1, 2, 3, 8, and 10), only NHE3, NHE8, and NHE10 are known to directly affect male fertility [37,104]. NHE3 is expressed on the apical membrane of epithelial cells in the efferent and epididymal ducts [105]. NHE8 is detected in the cytoplasm, co-localized with the Golgi apparatus in the Leydig cells, and in the developing acrosome of spermatids in the testis in mice [104,106]. NHE10 is the spermatozoa-specific NHE isoform [38]. NHE3KO mice have increased fluid volume in the testis with diminished sperm concentration, which leads to subfertility [107], while NHE10KO mice are infertile due to diminished sperm motility [38].

In NHE8KO mice, infertility is due to a lack of mature sperm in the seminiferous tubules and in the epididymis [104]. Adult NHE8KO male mice have small testis, and low testosterone levels, but normal LH and FSH serum levels. However, LHR protein is approximately 50% reduced in NHE8KO mice compared with wild-type mice. In NHE8 siRNA-transfected Leydig cells (MLTC-1), the expression of LHR is reduced to 35%. Upon stimulation with LH, the cAMP production, which is involved in the production of testosterone in the Leydig cells [108], is only 57% in siRNA-treated Leydig cells compared with control cells [104]. Besides the involvement of NHE8 in testosterone production, NHE8 also contributes to male fertility by affecting spermatogenesis. Karina Oberheide et al. showed that in the absence of NHE8, male mice are infertile due to a disruption of the acrosome formation, producing round-headed spermatozoa lacking the acrosomal cap, which resembles human globozoospermia [106].

## 6. NHE8 in Other Organs

As a ubiquitous protein, NHE8 is broadly expressed in different organs as represented by the illustration in Figure 1. Besides what we have discussed, NHE8 expression in mice is also detected in other tissues, such as the liver, lung, spleen, and skeletal muscle [90,109]. In humans, the expression of NHE8 is detected in almost all tissues [110]. Recently, NHE8 was localized in the apical membrane of alveolar epithelial cells in the lung, and its expression is subject to the regulation of angiotensin II. However, the precise role of NHE8 in those cells was not elucidated [111].

## 7. Conclusions

As evidenced in this review article, the function of NHE8 extends beyond a simple Na^+^/H^+^ exchanging transporter. Many discoveries have been made by different research groups showing the relevance of NHE8 in different organs. As part of the scientific community, our group has contributed enormously with findings involving the role of NHE8 in the eye, the testis, and the intestine. At present, NHE8 has been well-studied in the colon to show it contributes to mucosal protection by stimulating mucin production, which may be a pivotal molecule for goblet cell differentiation/maturation. However, the mechanisms involved in such processes are still unknown. Therefore, a more advanced investigation of this specific NHE isoform will be needed to discover its role during tissue development/differentiation.

## Figures and Tables

**Table 1:** The cation proton antiporter 1 (CPA1) superfamily.

Subdivision	Coding Gene	Molecular Weight	Inhibitors	Localization	Function	Ref.
NHE1	SLC9A1	820aa, ~90 kDa	Amiloride, EIPA, HMA, DMA, HOE-694, SM-20550, S-3226, Clonidine, Cimetidine	It is ubiquitously expressed in nearly all mammalian tissue types and localized basolaterally in every epithelial cell, except in the choroid plexus and syncytio-throphoblast of the human placenta where it expresses apically	It regulates cell volume, intracellular pH, proliferation, differentiation, and apoptosis	[8,13,14,15,16]
NHE2	SLC9A2	812aa, ~90 kDa	Amiloride, EIPA, DMA, S-3226	Highly expressed in intestine and kidney	The physiologic role of NHE2 still remains elusive despite its wide expression pattern with higher expression levels in the intestine and the kidney	[16,17,18,19]
NHE3	SLC9A3	834aa, ~92 kDa	Amiloride, Tenapanor, HMA, DMA, HOE-694, Zoniporide, S-3226, Clonidine, Cimetidine	Highly expressed in intestine and kidney	It plays an important role in mediating intestinal electroneutral NaCl absorption in mammals. It also contributes to the absorption of other nutrients, such as amino acids and dipeptides through H^+^ gradients	[8,16,19,20,21,22]
NHE4	SLC9A4	798aa, ~70 kDa	Amiloride, EIPA	NHE4 is highly expressed in the stomach while its expression is low in kidney, intestine, brain, ureter, skeletal muscle, heart, liver, and spleen	It plays a major role in intracellular pH homeostasis	[16,19,23,24]
NHE5	SLC9A5	896aa, ~100 kDa	Amiloride, EIPA, HMA, HOE-694, Cimetidine	Predominantly expressed in brain	It regulates growth factor signaling, integrin trafficking, and degradation of glioma cells. It also functions as a negative regulator of dendritic spine growth	[16,19,25,26]
NHE6	SLC9A6	669aa, ~85 kDa	Rimeporide	It is highly expressed in skeletal muscles, brain, and heart	It functions to regulate intramitochondrial Na^+^ and H^+^ gradients	[19,27,28,29,30]
NHE7	SLC9A7	725aa, ~80 kDa	Amiloride	Predominantly localized in the *trans*-Golgi network	It is prominently expressed in the trans-Golgi network and in the mid-trans-Golgi stacks, and functions as a K^+^/H^+^ exchanger in controlling organelle volume through transmembrane K^+^ flux	[16,19,31,32]
NHE8	SLC9A8	581aa, ~65 kDa	HOE-642, S-3226	NHE8 protein has been detected in the plasma membrane, Golgi apparatus, and endosomes. It is ubiquitously expressed in many tissues	NHE8 is involved in electroneutral NaCl transport, cell volume regulation, cellular adhesion, migration, and proliferation	[6,7,9,19,33,34]
NHE9	SLC9A9	645aa, ~72 kDa	Unknown	It is expressed mostly in heart, skeletal muscle, and brain	It transports the protons out of the endosomal lumen in exchange for Na^+^/K^+^, thus controlling the pH of endosomal lumens	[35,36]
NHE10	SLC9C1	1177aa, ~135 kDa	Unknown	Predominantly expressed in testis and in osteoclasts	NHE10 is associated with sperm motility and fertility, and it is required for osteoclasts differentiation and survivor	[19,37,38,39,40,41]
NHE11	SLC9C2	1124aa, ~130 kDa	Unknown	Expressed in the brain, choroid plexus, testis, and uterine tube	Unknown	[19]
NHA1	SLC9B1	515aa, ~55 kDa	Unknown	Expressed in testis	It regulates the pH and the cell volume	[19,42]
NHA2	SLC9B2	537aa, ~57 kDa	Phloretin	It is ubiquitously expressed in mouse tissues	It regulates the pH and the cell volume	[19,43,44]

EIPA = 5-N-ethyl-N-isopropyl-amiloride; HMA = 5-N,N-hexamethylene-amiloride; DMA = 5-N,N-dimethyl-amiloride; HOE-694 = 3-methylsulphonyl-4-piperidinobenzoyl, guanidine hydrochloride; SM-20550 = N-aminoiminomethyl-1,4-dimethyl-1H-indole-2-carboxamide methanesulfonic acid; S-3226 = 3-2-3-guanidino-2-methyl-3-oxopropenyl-5-methyl-phenyl-N-isopropylidene-2-methyl-acrylamide dihydrochloride.
